# Systemic Vascular Parameters in Ocular Pseudoexfoliation

**DOI:** 10.7759/cureus.62933

**Published:** 2024-06-22

**Authors:** Vaishnavi R Patil, K. Vallabha, Keerti Wali

**Affiliations:** 1 Ophthalmology, Shri B M Patil Medical College, BLDE, Vijayapura, IND

**Keywords:** exfoliation glaucoma, diabetes and hypertension, diabetes mellitus in elderly, senile cataract, anterior segment, ophthalmology

## Abstract

Background

Pseudoexfoliation syndrome (PEX) is characterized by a dandruff-like substance in the anterior chamber, composed of various glycoproteins that have an unclear origin. Its deposition is observed on the pupillary margin, lens zonules, and trabecular meshwork. Proteomic studies have identified numerous proteins in the affected individuals, suggesting associations with systemic conditions like heart disease, stroke, and Alzheimer’s disease. However, the systemic associations of PEX remain inconclusive, particularly in regions like southern India.

Materials and methods

A cross-sectional study was conducted on 114 participants. Pseudoexfoliation was graded as mild, moderate, and severe as per standard photographic grading. Systemic examinations included blood pressure measurements, electrocardiography (ECG), and blood investigations for serum lipid profile, fasting and postprandial blood sugar levels, and serum C-reactive protein levels. Small incision cataract surgery was performed for all the patients. Intraoperative complications and postoperative status were recorded.

Results

Thirty-eight patients (33.3%) had mild PEX, 44 (38.6%) had moderate PEX, and 32 (28.1%) had severe PEX. Hypertension was present in 54 participants (47.4%), diabetes in 21 (18.4%), coronary artery disease in nine (7.9%), and cerebrovascular accidents in three (2.6%). The mean systolic blood pressure was 140.39 mmHg and the mean diastolic blood pressure was 90.37 mmHg. Systolic blood pressure exceeded 140 mmHg in 29 participants (90.6%) with severe PEX, while diastolic blood pressure surpassed 90 mmHg in 26 participants with severe PEX, both with a p-value of 0.001. Mean fasting and postprandial blood sugar levels were 103.80 ± 31.81 mg/dl and 131.72 ± 48.24 mg/dl, respectively. Serum lipid profiles showed mean low-density lipoprotein (LDL), very low-density lipoprotein (VLDL), cholesterol, and triglyceride levels of 103.00 ± 34.49 mg/dl, 29.04 ± 15.51 mg/dl, 172.73 ± 43.34 mg/dl, and 129.33 ± 64.65 mg/dl respectively. Electrocardiographic results indicated that 54 participants (47.37%) had abnormal ECG including rate abnormality in 13.2%, conduction defects in 12.3%, ischemic changes in 10.5%, and structural defects in 11.4%. Eighty-seven percent of patients had non-dilating pupils and iris atrophy, 13.2% had zonular dialysis and intraoperatively, 78% had capsulorhexis extension, 49.12% had difficult nucleus prolapse, and 28.95% had posterior capsular rent.

Conclusion

This study highlights the significantly elevated parameters of systemic vascular diseases in PEX patients, like elevated blood pressure and more frequent cardiac anomalies, emphasizing the need for comprehensive systemic evaluation and careful preoperative assessment for ocular comorbidities.

## Introduction

Pseudoexfoliation (PEX) syndrome was first documented by John Gustaf Lindberg almost a hundred years ago [1,2]. It is characterized by a ‘dandruff-like’ substance in the anterior chamber with a unique deposition pattern on the anterior lens capsule, creating a double concentric ring pattern with a clear zone associated with an iris movement. PEX material is also observed at the pupillary margin, lens zonules, and trabecular meshwork. It is composed of various glycoproteins with an unclear origin, potentially deriving from the iris, lens epithelium, ciliary body, or trabecular meshwork [3]. PEX has a significant variation in prevalence across different geographical regions, ranging from 0% to 38%, even within the same population, and its prevalence increases with age [4,5]. Several genes have been attributed to PEX, notably the LOX1 and CLU genes [6-9].

Proteomics studies have identified proteins such as vitamin D-binding protein, apolipoprotein A4, lysyl oxidase-like-1, and complement C3 in the anterior segment tissues and fluids of individuals affected by PEX [10]. The protein composition of PEX syndrome was investigated using liquid chromatography and tandem mass spectrometry, identifying 66 proteins, including 13 novel constituents, with gene expression analysis and pathway studies implicating extracellular matrix organization, elastic fiber formation, and the complement cascade, offering molecular insights into the disease’s complexity and its associations with heart disease, stroke, and Alzheimer’s disease [11,12].

Electron microscopic and ultrastructural examinations have demonstrated the presence of PEX materials in various visceral organs and extraocular sites, identifying them adjacent to elastic and oxytalan fibers, with positive staining for elastin and human amyloid P protein, resembling characteristics observed in ocular sites [3,13,14]. Moreover, there has been evidence of coronary artery disease, hypertension, electrocardiogram abnormalities, dyslipidemia, and elevated C-reactive protein in PEX patients [15-21].

Substantial literature suggests a positive association of PEX with ischemic heart disease and blood pressure, limited by reliance on hospital health records for systemic disease assessment [19,22-24]. Few studies have been conducted on PEX and its systemic associations in India. Due to inconclusive and inconsistent results in previous studies, further research is needed, especially in southern India. It may have significant public health and clinical implications, as slit-lamp examinations for PEX diagnosis could identify individuals at increased risk of diabetes, hyperlipidemia, hypertension, and cardiovascular abnormalities. This study aims to determine the correlation between severity of ocular PEX and abnormalities of systemic vascular parameters.

## Materials and methods

A cross-sectional study was conducted at a tertiary care hospital from September 2022 to August 2023. Out of 6120 screened patients, 216 were identified with ocular PEX, and 114 met the inclusion and exclusion criteria for the study. Patients aged over 40 years with ocular PEX and cataracts were included in the study. Individuals with a history of prior intraocular surgery, a history of trauma or uveitis, significant corneal opacities that obscure anterior segment structures, or any ocular pathology that could result in secondary glaucoma were excluded from the study.

**Figure 1:**
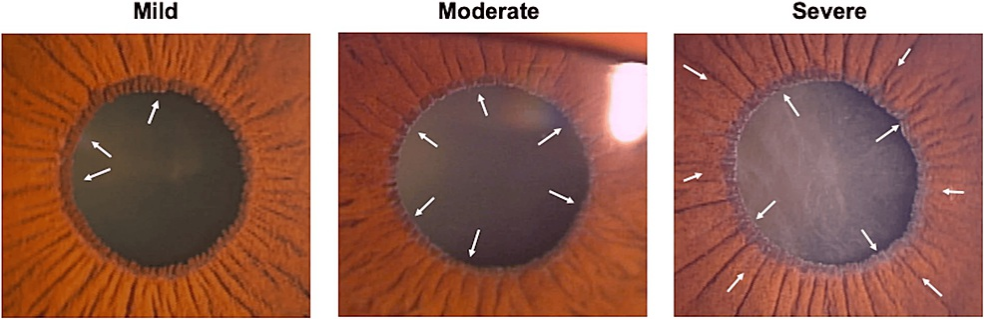
Photographic grading of pseudoexfoliation Reproduced with the permission of Aoki et al. [25].

After informed consent, a detailed patient history was taken, including history of trauma and prior intraocular surgeries. A history of systemic comorbidities was noted, including hypertension, diabetes, coronary artery disease (CAD), and stroke. The best-corrected visual acuity was evaluated using a Snellen chart. Slit lamp examination (AIA 11-5S-L; Appasamy Associates, Chennai, India) was done after diagnostic mydriasis with a drop of 1% tropicamide. Ocular PEX was diagnosed based on the presence of characteristic greyish-white exfoliation material on the anterior capsular surface of the lens, manifesting in various forms such as a complete or partial peripheral band, a central shield, and extending to other areas in the anterior chamber, along with pre-capsular frosting or haze. All participants were graded into three groups (mild, moderate, and severe) as per the standard slit-lamp photographic grading explained by Aoki et al. [25] (Figure 1).

Intraocular pressure was measured using a Goldmann applanation tonometer (AATM 5001; Appasamy Associates, Chennai, India). Gonioscopy was performed with a four-mirror goniolens (MIPL/14; Opticlear Ophthalmic Lenses, New Delhi, India), and fundus examination was carried out using binocular indirect ophthalmoscopy (AIO-7; Appasamy Associates, Chennai, India). Systemic examination was performed. Blood pressure was measured with a well-calibrated mercury sphygmomanometer in the left arm in a supine position. The starting of the first Korotkoff sound was taken as systolic blood pressure, and the ending of the fourth Korotkoff sound was considered diastolic blood pressure. Blood pressure was measured three times at one-hour interval and an average of three measurements was taken.

Twelve-lead electrocardiography (Cardiart 6208, BPL, Bengaluru, India) was performed, and the interpretation of the electrocardiogram (ECG) was graded into five groups (Table 1).

**Table 1:** Groups of different electrocardiogram findings Author's own creation.

Group	Electrocardiogram findings
1	Normal
2	Rate defects
3	Conduction defects
4	Ischemic defects
5	Structural defects

Blood investigations included serum lipid profile, fasting and postprandial blood sugar levels, and serum C-reactive protein levels, estimated in an automated analyzer (VITROS 5.1FS; Ortho-Clinical Diagnostics Inc., Raritan, NJ, USA). A plain bulb was used for lipid profile and C-reactive protein and a grey bulb containing sodium fluoride was used for fasting and postprandial blood sugar levels. Early morning fasting blood samples were collected to measure fasting blood sugar (FBS) levels, serum low-density lipoprotein (LDL), serum very low-density lipoprotein (VLDL), serum triglyceride, serum cholesterol, and serum C-reactive protein (CRP) levels. The blood sample for postprandial blood sugar (PPBS) level analysis was collected two hours after breakfast. 

All patients underwent small incision cataract surgery with intraocular lens implantation wherever possible. Preoperatively, 1% tropicamide eye drops were instilled for pupillary dilation along with flurbiprofen 0.03% eye drops for pupillary stabilization. The intraoperative and postoperative course was recorded for every patient.

Statistical analysis

The collected data was entered into a Microsoft Excel sheet (Office 365 suite, Microsoft, Redmond, WA), and statistical analysis was conducted using the SPSS Statistics Version 20 (IBM Corp., Armonk, NY). The results are expressed as mean (median) ± SD, counts, and percentages and represented using diagrams. The association of systemic parameters with the grade of PEX was analyzed using the chi-square test.

## Results

**Figure 2:**
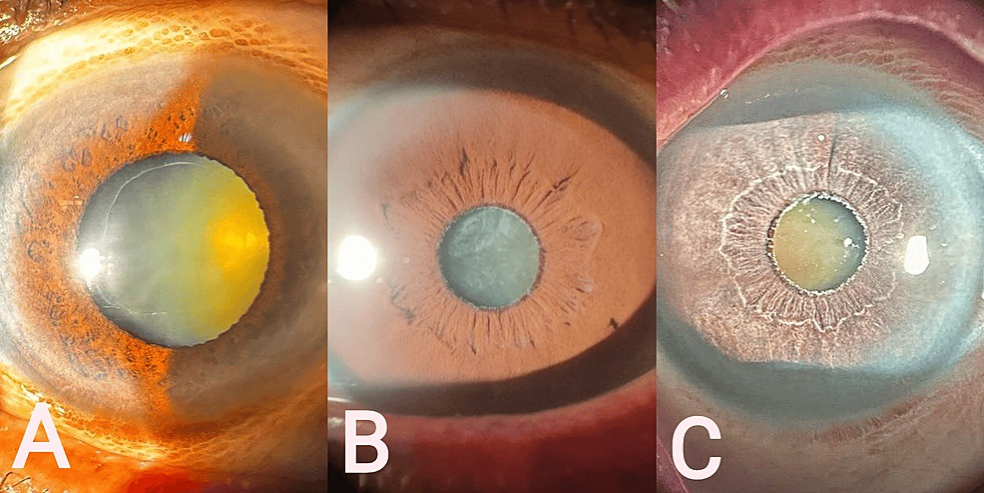
PEX eyes as seen during the study. A: mild PEX with maximum pupillary dilatation; B: moderate PEX; C: severe PEX. PEX: Pseudoexfoliation

In this study, 114 participants were included. The participants had a mean age of 68.95 ± 8.07 years, with 64 (56.1%) males and 50 (43.9%) females. Thirty-eight (33.3%) had mild PEX, 44 (38.6%) had moderate PEX, and 32 (28.1%) had severe PEX. Out of the participants, 47.4% of participants were hypertensive, 18.4% had diabetes, 7.9% had a history of coronary artery disease, and 2.6% had a history of cerebrovascular accidents (Table 2 and Figure 2).

**Table 2:** Baseline characteristics SD: Standard deviation

Baseline characteristics	Frequency (n)	Percentage (%)
Age in years (mean ± SD)	68.95 ± 8.07	
Gender		
Male	64	56.1
Female	50	43.9
Laterality		
Both eyes	72	63.2
Right eye	24	21.1
Left eye	18	15.8
Grading of pseudoexfoliation		
Mild	38	33.3
Moderate	44	38.6
Severe	32	28.1
Systemic comorbidities		
Hypertensive	54	47.4
Diabetic	21	18.4
Coronary artery disease	9	7.9
Stroke	3	2.6

Participants' mean systolic blood pressure was 140.39 mmHg and their mean diastolic blood pressure was 90.37 mmHg. The average fasting blood sugar was 103.80 mg/dl and postprandial blood sugar was 131.72 mmHg. Mean serum LDL, VLDL, cholesterol, and triglyceride values were 103 mg/dl, 29.04 mg/dl, 172.73 mg/dl and 129.33 mg/dl, respectively (Table 3).

**Table 3:** Systemic and blood parameters LDL: Low-density lipoprotein VLDL: Very low-density lipoprotein

Systemic and blood parameters	Mean ± standard deviation
Systolic blood pressure (mmHg)	140.39 ± 12.54
Diastolic blood pressure (mmHg)	90.37 ± 11.05
Fasting blood sugar (mg/dl)	103.80 ± 31.81
Postprandial blood sugar (mg/dl)	131.72 ± 48.24
Serum LDL (mg/dl)	103.00 ± 34.49
Serum VLDL (mg/dl)	29.04 ± 15.51
Serum cholesterol (mg/dl)	172.73 ± 43.34
Serum triglyceride (mg/dl)	129.33 ± 64.65

Sixty participants (52.6%) had normal ECG findings and the rest 54 (47.4%) of the participants had abnormal ECG findings (Table 4).

**Table 4:** Distribution of ECG changes ECG: Electrocardiogram.

ECG changes	No. of participants	Percentage (%)
Normal	60	52.6
Rate defect	15	13.2
Conduction defect	14	12.3
Ischemic changes	12	10.5
Structural defects	13	11.4

Eighty-seven percent of participants had non-dilating pupils and iris atrophy, while 13.2% had zonular dialysis. Intraoperatively, 78% had an extension of capsulorhexis, 49.12% had a difficult nucleus prolapse, and 28.95% had posterior capsular rent (Table 5).

**Table 5:** Ocular complications of pseudoexfoliation

Complications	Frequency [n]	Percentage [%]
Ocular complications		
Non-dilating pupil and iris atrophy	99	86.8
Zonular dialysis	15	13.2
Intraoperative complications		
Extension of capsulorhexis	78	68.4
Difficulty in nucleus prolapse	56	49.1
Posterior capsular rent	33	29.0
Aphakia	8	7.0
Nucleus drop	1	0.9

Thirty-eight participants had mild PEX, 44 had moderate PEX, and 32 had severe PEX. Systolic and diastolic blood pressure showed a significant association with the severity of PEX (p value=0.001, chi-square test) unlike all other parameters (Table 6).

**Table 6:** Association of grades of PEX with different systemic and electrocardiogram parameters SBP: Systolic blood pressure. DBP: Diastolic blood pressure. LDL: Low-density lipoprotein. *P value < 0.05 is considered as statistically significant.

Systemic parameter	Severity of PEX			P-value
	Mild (n = 38)	Moderate (n = 44)	Severe (n = 32)	
SBP more than 140 mmHg	03 (7.9%)	20 (45.5%)	29 (90.6%)	0.001***
DBP more than 90 mmHg	03 (7.9%)	19 (43.2%)	26 (81.3%)	0.001***
Fasting blood sugar	03 (7.9%)	06 (13.6%)	09 (28.1%)	0.061
Postprandial blood sugar	02 (5.3%)	03 (6.8%)	05 (15.6%)	0.263
Serum cholesterol more than 200 mg/dl	09 (23.7%)	11 (25.0%)	06 (18.8%)	0.804
Serum LDL more than 130 mg/dl	10 (26.3%)	08 (18.2%)	05 (15.6%)	0.494
Serum triglyceride more than 150 mg/dl	08 (21.1%)	10 (22.7%)	11 (34.4%)	0.386
C-reactive protein more than 10 mg/dl	02 (5.3%)	04 (9.2%)	04 (12.5%)	0.564
Electrocardiogram findings				
Normal	25 (65.8%)	23 (52.3%)	12 (37.5%)	0.418
Rate defects	05 (13.2%)	05 (11.4%)	05 (15.6%)	
Conduction defects	02 (5.3%)	07 (15.9%)	05 (15.6%)	
Ischemic defects	04 (10.5%)	04 (9.1%)	04 (12.5%)	
Structural defects	02 (5.3%)	05 (11.4%)	06 (18.8%)	

## Discussion

The study aimed to investigate the correlation between the severity of ocular PEX and systemic vascular diseases and identify perioperative complications of PEX syndrome. We considered systemic vascular parameters like systolic and diastolic blood pressure, glycaemic status, dyslipidemia and cardiac anomalies as detected on electrocardiogram. We found elevated systolic and diastolic blood pressure in patients with PEX. We observed an increased percentage of ECG abnormalities. The mean age of the study participants was 68.95 years (Table 1). In the Vardhan et al. study [19], the mean age was 64.8 years, while in the study by French et al., the mean age was 77.1 years [26]. As per the study by Young et al., most patients were over 65 years old [27]. Vardhan et al.'s study had 50.5% males and 49.5% females, comparable to our study (56.1% males and 43.9% females) [19] (Table 1). However, Young et al. studied a Chinese cohort, noting a higher percentage of female participation of 63% [27].

In this study, we found 47.4% of patients to be hypertensive (Table 1), which was interestingly higher compared to the study by Pooja et al., who reported 14.92% of the participants to be hypertensive [22]. However, the percentage of people with diabetes was comparable to our study. We reported that 25.37% of the participants had diabetes (Table 1), and Pooja et al. noted patients with diabetes at 18.4% in their study [22].

Our study reported 7.9% of patients having a history of coronary artery disease and 2.6% of patients with a history of cerebrovascular accidents (Table 1). In comparison, Imaz Aristimuño et al. reported that 3.7% of the patients had coronary artery disease patients and 4.6% were stroke patients in their study [28].

Vardhan et al. reported mean systolic blood pressure (SBP) to be 131.8 mmHg and diastolic blood pressure (DBP) to be 78.1 mmHg [19]. Meanwhile, Akarsu et al. reported 120 mmHg mean SBP and 80.5 mmHg mean DBP [29]. In our study, the mean SBP was 140.39 ± 12.54 mmHg, and the mean DBP was 90.37 ± 11.05 mmHg, with a statistically significant association between blood pressure and the severity of PEX. Špečkauskas et al. observed a statistically significant increased prevalence of arterial hypertension in PEX compared to non-PEX without any comparison to PEX severity [30].

Vardhan et al. compared random blood sugar levels in patients with PEX and without PEX and reported it to be 125.2 (±68.3) mg/ml and 119.2 (±51.6) mg/ml in PEX and non-PEX groups [19]. We estimated the mean fasting blood sugar (FBS) to be 103.80 ± 31.81 mg/dl and the mean post-prandial blood sugar (PPBS) to be 131.72 ± 48.24 mg/dl. However, the association with PEX severity is not statistically significant.

Mitchell et al. [31] and Citirik et al. [32] reported a positive correlation of PEX with coronary artery disease. However, they did not explain the association with any specific coronary defect [31,32]. Vardhan et al. revealed that the incidence of left ventricular hypertrophy (LVH) was higher in the PXF patients than in the controls (p = 0.02) [19]. In this study, 54 patients (47.37%) had an abnormal ECG finding, 13.2% had rate defects, 12.3% had conduction defects, 10.5% had ischemic changes, and 11.4% had structural defects. There was no significant association between the severity of PEX and different electrocardiogram findings (Table 6).

We also did not find significantly elevated CRP among PEX cases, and CRP level was not associated with the severity of PEX. Our findings aligned with the studies by Sorkhabi et al. [18], Lesiewska et al. [33], Yüksel et al. [34] and Kymionis et al. [35].

Preoteasa et al. reported 156 perioperative incidents in 999 eyes with pseudoexfoliation, with predictive factors identified as a shallow anterior chamber, cataract grade, preoperative intraocular pressure, and symmetry of the exfoliation material [36]. Similar reports were also mentioned by Vazquez-Ferreiro et al. [37]. Our study noticed major intraoperative complications: capsulorhexis extension, difficult nucleus delivery and posterior capsular rent.

The limitations of our study are a comparably smaller sample size and not comparing our data with non-PEX counterparts. We recommend further studies to address these gaps.

## Conclusions

This study highlights significantly deranged parameters of systemic vascular diseases in PEX participants. Participants with PEX exhibited elevated systolic and diastolic blood pressure, which increased with the severity of PEX, and more frequent cardiac anomalies as detected by ECG. These findings indicate the importance of comprehensive systemic evaluation in participants with PEX, particularly in older populations. The ocular complications associated with PEX, such as non-dilating pupils, iris atrophy, and intraoperative challenges during cataract surgery, also emphasize the need for careful preoperative assessment and planning. The study calls for further research to explore the systemic implications of PEX and to develop strategies for early detection and management of associated conditions, particularly in regions with limited data, such as southern India. Early identification of PEX through slit-lamp examinations can be critical in recognizing individuals at an increased risk of systemic diseases, potentially improving public health outcomes and clinical care for affected individuals. Further studies can explore how artificial intelligence can be utilized with anterior segment imaging to detect pseudoexfoliation and screen for systemic diseases.
